# Liver Specification in the Absence of Cardiac Differentiation Revealed by Differential Sensitivity to Wnt/β Catenin Pathway Activation

**DOI:** 10.3389/fphys.2019.00155

**Published:** 2019-03-05

**Authors:** Kim Haworth, Lee Samuel, Sarah Black, Pavel Kirilenko, Branko Latinkic

**Affiliations:** School of Biosciences, Cardiff University, Cardiff, United Kingdom

**Keywords:** Gata4, *Xenopus*, liver, heart, Wnt, FGF, BMP

## Abstract

Embryonic precursors of liver and heart, whilst not sharing cellular origin, develop in close proximity through a dynamic series of inductive signaling events. During gastrulation anterior endoderm (AE) provides cardiogenic signals that act on adjacent mesoderm, resulting in induction of cardiac precursors. Subsequently cardiogenic mesoderm generates a FGF signal that acts on adjacent AE to induce foregut organ specification. Additional signals such as BMP and Wnt provide further information required for liver specification. Most findings on liver specification were derived from mouse explant studies as well as experiments with *Xenopus* and zebrafish embryos. To address some of the limitations of these models, here we used two complementary *ex vivo* models based on *Xenopus* embryos: pluripotent animal cap explants expressing Gata4 transcription factor and conjugates of gastrula-stage AE with animal caps (AC). We show that in these models liver specification is not sensitive to Wnt signaling manipulation, in contrast to the requirement for Wnt antagonism shown *in vivo*. FGF pathway is not necessary for Gata4-induced liver specification in animal cap explants but is required for prolonged period in sandwiches of AE and AC. In contrast, BMP signaling is shown to be essential for Gata4-induced liver specification. Our findings may have implications for research on liver differentiation from embryonic stem cells.

## Introduction

During embryonic development liver is induced in foregut endoderm by diverse and dynamic signaling from surrounding mesodermal tissue. Classical embryological experiments in the avian model have identified cardiac mesoderm as a source of an essential signal that specifies liver primordium induction and outgrowth from the adjacent gut tube (Zaret, 2008, 2016; Zorn and Wells, 2009). These findings were confirmed in the mouse explant system, which reconstitutes interactions between cardiogenic mesoderm and ventral endoderm (Gualdi et al., 1996). The mouse explant assay has been used to identify FGF signaling as a cardiac mesoderm-derived factor that induces liver-specific gene expression (Jung et al., 1999). Furthermore, BMP derived from the adjacent septum transversum mesenchyme was shown to be required together with FGF for liver specification (Rossi et al., 2001). In addition to FGF and BMP signaling, the Wnt pathway has been implicated in liver specification (McLin et al., 2007; Gordillo et al., 2015; Zaret, 2016). The actions of these signaling pathways in early liver development are highly dynamic and dose-dependent.

The close relationship between embryonic liver and heart likely begins early in development, during gastrulation. Experimental evidence from chick and frog models have suggested that during gastrulation dorso-anterior endoderm, a tissue that will contribute to the liver formation, is required to induce cardiac tissue in adjacent mesoderm (Lough and Sugi, 2000). Later on, after hepatic specification, signals arising from developing liver bud appear to induce the formation of the proepicardium in the mesothelium in later cardiac development (Ishii et al., 2007). Therefore, the fates of developing heart and liver may be tied by several rounds of reciprocal signaling.

Liver-inducing signals regulate the transcriptional program in foregut endoderm via pioneer transcription factors FoxA and Gata4 which have the ability to associate with target genes in compacted heterochromatin (Zaret, 2016). Gata4 and Gata6 zinc-finger transcription factors have conserved roles in liver development in the mouse, zebrafish, and frog (Gordillo et al., 2015). In addition, Gata5 has been shown to regulate liver development in *Xenopus* (Haworth et al., 2008). The Gata4/5/6 family of transcription factors have well-documented roles in other tissues, notably the heart (Charron and Nemer, 1999).

Of relevance for the current study, Gata4, a hepatic pioneer transcription factor, has cardiogenic activity: gain of function of Gata4 alone, or together with other cardiac factors, can induce cardiogenesis in *Xenopus* and mouse embryos, respectively (Latinkic et al., 2003; Takeuchi and Bruneau, 2009). In pluripotent animal pole cells from *Xenopus* blastula embryos Gata4 induces not just cardiac cell fate, but also liver cell fate (Latinkic et al., 2003). This finding provides an experimentally amenable model of co-induction of cardiac and liver fates to study the mechanisms involved.

We have complemented the Gata4-based induction model with another *Xenopus* model developed for investigating the inductive capacity of anterior endoderm (AE) (Samuel and Latinkic, 2009). In this model, early gastrula anterior endoderm explants are conjugated with pluripotent responding tissue, blastula stage animal caps (AC). AC/AE conjugates were shown to recapitulate cardiogenic signaling between the source, AE, and the responder, AC (Samuel and Latinkic, 2009). Here we show that AC/AE closely mimic cellular and molecular interactions as they occur during liver induction as well. AE explants in isolation retain endodermal characteristics but fail to adopt liver fate, which can be induced if AE is conjugated with AC tissue. An AE-derived signal first induces cardiac precursors in AC, which appear to generate a signal that acts on AE to induce liver cell fate.

Using both the Gata4 and AC/AE models, we show that active Wnt signaling is compatible with hepatic specification despite the well-known inhibitory effect on cardiac differentiation. In addition, we show that Gata4 induces liver cell fate independently of FGF signaling but requires BMP signaling.

## Materials and Methods

### Embryos and Explants

All work with *Xenopus laevis* (obtained from Nasco or raised in our facility) was approved by Cardiff University’s Ethical Review Committee and was undertaken under a license from the United Kingdom Home Office. *Xenopus laevis* embryos were obtained by mating of frogs primed with human chorionic gonadotrophin (Sigma; 700 units per female and ∼150 units per male) or by *in vitro* fertilization (Sive et al., 2000). Jelly membrane was removed with 2% cysteine-HCl, ph7.8 (Sive et al., 2000). Embryos were grown in 10% Normal Amphibian Media (NAM) and staged as described (Sive et al., 2000). AC and AE explants were carried out in 75% NAM as described (Samuel and Latinkic, 2009). Typical samples had 12–20 AC/AE explants and 25–30 ACs. AC/AE experiments and gel RT-PCR analysis of AC experiments were repeated at least twice. Whole embryos (WE) or explants were cultured until age match control siblings had reached desired stage. Micronjections were carried using an IM 300 Micro-injector (Narishige Scientific), in 75% NAM containing 3% Ficoll (Sigma). Morpholino Oligonucleotides (MOs) were supplied from Gene Tools^1^ and injected at 10 nl/embryo. *Cerberus* antisense morpholino oligomer (CerMO) (Kuroda et al., 2004), *hhex*MO (Smithers and Jones, 2002), and Control MO (Haworth et al., 2008) were injected as described (Haworth et al., 2008). 20 ng/embryo of Control MO was injected (Supplementary Figure S5). mMESSAGE mMACHINE kit (Ambion) was used for capped mRNA synthesis. Templates used have been previously described: Gata4 (Gallagher et al., 2012), Cerberus (Bouwmeester et al., 1996), *dkk-1* (Glinka et al., 1998), dominant-negative FGFR1 (XFD; Amaya et al., 1991), dominant-negative BMPR (tBR; Graff et al., 1994), LEF-β-GR (Domingos et al., 2001), *sox17* (Hudson et al., 1997), *hhex*-VP2 (Brickman et al., 2000), and CSKA-Wnt8 DNA (Smith and Harland, 1991) and were injected at 100 pg/embryo. Injection solutions included lineage tracers biotin- and rhodamine-dextran (Invitrogen; Latinkic et al., 2003). LEF-β-GR was induced by adding dexamethasone [DEX (Sigma); stored as 2 mM stock in ethanol] into embryonic media to a final concentration of 2 μM.

### Drug Treatment

SU5402 (Calbiochem; Mohammadi et al., 1998) and Dorsomorphin (Sigma; Yu et al., 2008) were dissolved in DMSO and used at indicated concentrations.

#### Gene Expression Analyses

Total RNA was isolated from samples using TRIzol reagent (Invitrogen) or the acid guanidinium thiocyanate-phenol-chloroform method (Chomczynski and Sacchi, 1987). cDNA was synthesized using MMLTV or RevertAid (Thermo Fisher Scientific) according to manufacturer’s instructions, using random hexamers. Approximately 1 μg of total RNA was used per sample. PCR was carried out using GoTaq polymerase (Promega, United Kingdom) according to manufacturer instruction. Primers were described (Samuel and Latinkic, 2009) except for: *nkx2-1*F 5′-tctcaggccagtatgcaaca; *nkx2-1* R 5′-cacttgagcctgggagaga (34 cycles); *insulin* F 5′-tgggtctcacctggtagaagc; *insulin* R 5′-tgggcaacattgctccacaatcc (36 cycles); *amy2a* F: 5′-cgtggcaagattgccgaatac; *amy2a* R 5′-ccattccatttgcggatgactc (36 cycles); *pdx1* F 5′-tgccattcccagatgacaacg; *pdx1* R 5′-ccttctctagttccagctg (35 cycles); *pdia2* F 5′-atttcaacaaggccctagagacc; *pdia2* R 5′-atcgatgtggcctgtttc (34 cycles); *fabp1* F 5′-accgagattgaacagaatgg; *fabp1* R 5′-cctccatgtttaccacggac (32 cycles) (AF068301); *fabp2* F 5′-tacccttgcacaaccctttg; *fabp2* R 5′-aatagatggcccgtcaggtc (32 cycles) (NM_001085877); *nr1h5* F 5′-agtgggaagatctggagca; *nr1h5* R 5′-tgcactgaacttcagtgagc (35 cycles). Quantitative PCR (Q-PCR) was performed on a Bio-Rad Mini Opticon MJ mini cycler using SYBR green fluorescent reagent. Samples were amplified in duplicate or triplicate and amplification of the endogenous reference gene *odc1* was performed in wells alongside target genes of interest. Primer pairs were described (Samuel and Latinkic, 2009) or were newly designed: *nr1h5* qF 5′-gagtatgcattactttcagcag; *nr1h5* qR 5′-tgttagacgtccaatcagtcga; *foxA2* qF 5′-gacacgaagctacagattggagc; *foxA2* qR 5′-ctcatgcccgtgttcacatagg; *sox17* qF 5′-gcagagcagatcacatccaa; *sox17* qR 5′-ttgtctgcagtaggcaccac. Ct values were determined and fold change relative to odc1 as described by Livak and Schmittgen (2001). Q-PCR data is shown in graphs with standard deviations and number of independent experiments (*n*) indicated. Repeated RT-PCR experiments showed that the data is semi-quantitative, providing good overall agreement with qPCR data (Samuel and Latinkic, 2009).

Double Whole Mount *in situ* Hybridization was performed as described (Sive et al., 2000; Haworth et al., 2008). Probes used were described: *myl7* (Chambers et al., 1994) and *nr1h5* (Seo et al., 2002).

Western blotting to detect exogenous (injected) Gata4 protein using HA tag was as described (Gallagher et al., 2012).

## Results

### Induction of Hepatogenesis in Gata4-Expressing Animal Cap Explants and Animal Cap/Anterior Endoderm Conjugates

**FIGURE 1:**
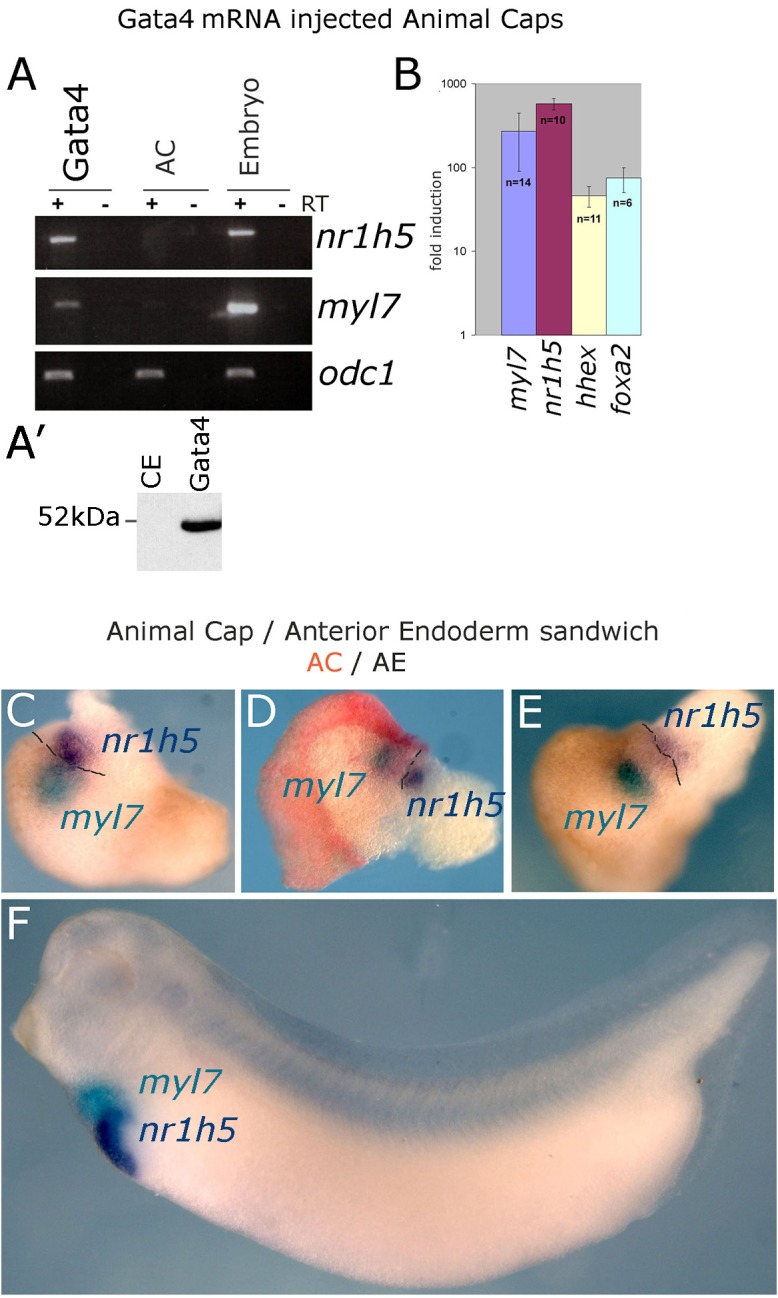
Induction of hepatogenesis in Gata4-expressing AC explants and AC/AE conjugates. **(A,B)** Marker gene analysis in Gata4-expressing animal caps. **(A)** RT-PCR showing expression of cardiac fate marker *myl7*, liver fate marker *nr1h5* and control marker *odc1* in Gata4 mRNA-injected ACs, control ACs and stage 33 sibling control embryo. Representative of four independent experiments. **(A′)** Western blotting shows efficient translation of exogenous Gata4 protein. An equivalent of ½ embryo at st. 9 from a sample of five embryos has been loaded on the gel. **(B)** qPCR showing fold difference in expression between Gata4 treated animal caps and control animal caps grown to st. 33, induction of expression of *myl7*, *nr1h5*, *hhex* and marker of endoderm *foxa2* is shown. Number of independent experiments (*n*) and standard error of the mean are shown. **(C–E)** Cardiac and liver domains are adjacent in AC/AE conjugates and **(F)** age matched sibling controls. AC/AE conjugates (sandwiches) were cultured until sibling control embryos reached st. 34 and were processed for *myl7* expression (turquoise) and *nr1h5* (purple) by WMISH. AC-derived tissue is shown in pink/red. Three AC/AE examples are shown demonstrating adjacent expression domains of *myl7* in the AC-derived tissue and nr1h5 in the AE-derived component of the explant (12/12 specimens showed the same pattern). Dashed line highlights the border between AC and AE.

Expression of transcription factor Gata4 in animal cap explants from blastula stage embryos has been shown to lead to cardiac differentiation (Latinkic et al., 2003). In this model Gata4 does not act with exclusive cardiac specificity but also induces early endoderm markers as well as a liver marker *fabp1* (formerly known as *lfabp*). High level of liver-specific expression of *fabp1* appears in late tadpole stages, beyond the practical limit of culturing AC explants. We have re-examined liver induction in Gata4 mRNA-injected AC explants by using expression of a liver-specific early tadpole marker *nr1h5* (formerly known as *for1*), as well as liver-enriched markers *hhex* and *foxa2*. Our analyses demonstrate that Gata4 induces liver cell fate in AC explants, in addition to cardiac cell fate (Figure 1A,B).

Gata4 induces cardiac and liver cell fates in uniformly injected AC explants (Latinkic et al., 2003), suggesting that under conditions of gain of function of Gata4 in pluripotent AC explants, liver cell fate is induced cell-autonomously and that fate acquisition is stochastic. This activity of Gata4 is consistent with its well-known roles in liver specification *in vivo* (Gordillo et al., 2015; Zaret, 2016). We further explored the question of cell autonomy of liver specification by endoderm specifiers and to that end we used Sox17, which in early vertebrate embryos is an exclusive endoderm determinant. Sox17 induces endoderm in AC explants, both when uniformly expressed and when expressed in half of each explant (Gallagher et al., 2014). Cardiac tissue is induced in hemi-injected explants only (Supplementary Figure S1; Gallagher et al., 2014). Similarly, liver marker *nr1h5* expression is induced only in hemi-injected *sox17* AC explants, strongly suggesting that liver cell fate is induced non-cell autonomously by a determinant of early endoderm (Supplementary Figure S1).

In addition to Gata4-triggered cardiogenesis in AC explants, we have developed a model that uses endogenous cardiogenic signal produced by the gastrula stage AE to induce cardiac cell fate in juxtaposed (conjugated) AC tissue (Samuel and Latinkic, 2009). AE explants express endodermal markers *a2m* and *sox17* as well as AE marker *hhex* (Supplementary Figure S2). During culturing period, AE explants retain endodermal characteristics (*a2m* and *sox17*) as well as *hhex* expression. Given that AE express *hhex*, which at tadpole stages marks both liver and endothelial cells, and that AC/AE conjugates contain endothelial cells (Samuel and Latinkic, 2009), the expression of *hhex* cannot be used to monitor liver specification in this model.

**FIGURE 2:**
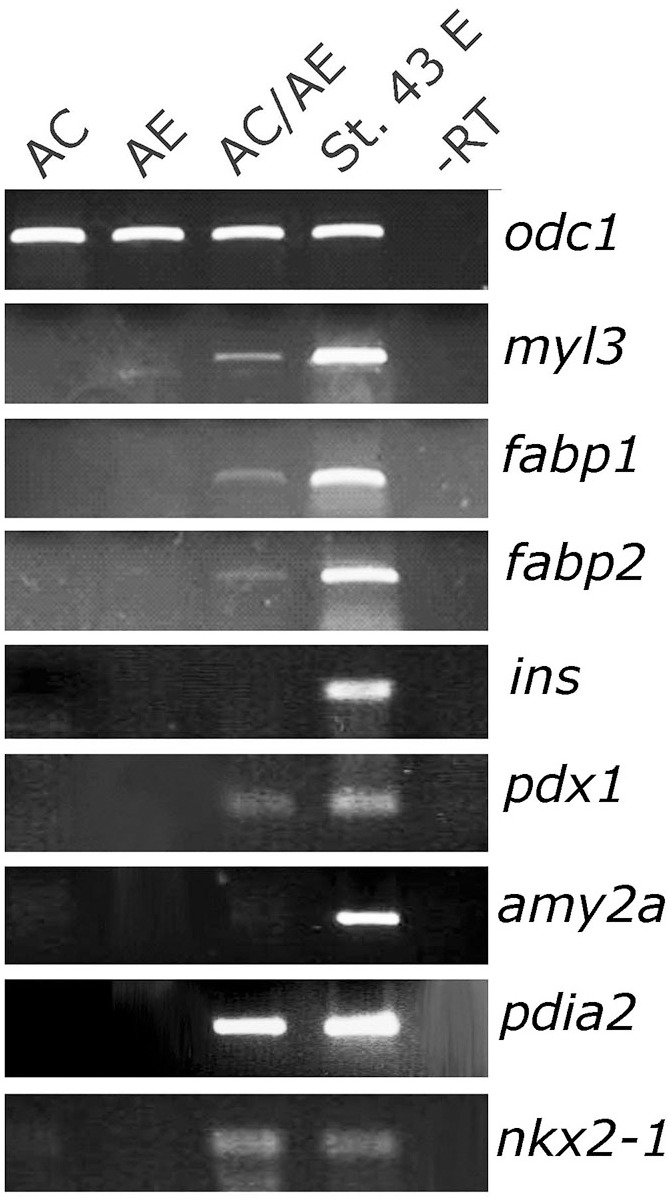
Endoderm diversification in AC/AE conjugates. Examination of gene expression in st. 43 AC/AE explants by RT-PCR reveals expression of liver (*fabp1*), intestine (*fabp2*), pancreas (*pdia2* and *pdx1*, but not *ins* and *amy2a*), thyroid/lung (*nkx2-1*) as well as ventricular cardiomyocyte (*myl3*) markers. St. 43 E-sibling control embryo at st. 43.

At tadpole stage (st. 34) AC/AE conjugates showed *nr1h5* expression in AE, adjacent to the domain of cardiomyocytes marked by *myl7*, in a manner resembling the close spatial relationship of the developing heart and liver in the embryo (Figure 1C–F). Upon prolonged culture until st. 43, the conjugates showed evidence of endodermal fate diversification, by expressing liver (*fabp1*), intestine (*fabp2*), pancreas (*pdia2* and *pdx1*), and lung/thyroid (*nkx2*-*1*) markers (Figure 2). Despite expressing *pdia2* and *pdx1*, AC/AE conjugates did not express *insulin* or *amy2a*, suggesting incomplete pancreatic reprogramming. As previously shown (Samuel and Latinkic, 2009), AC/AE expressed cardiac ventricular marker *myl3* as well.

### Cerberus and *hhex* Are Required in Anterior Endoderm for Liver and Cardiac Specification

**FIGURE 3:**
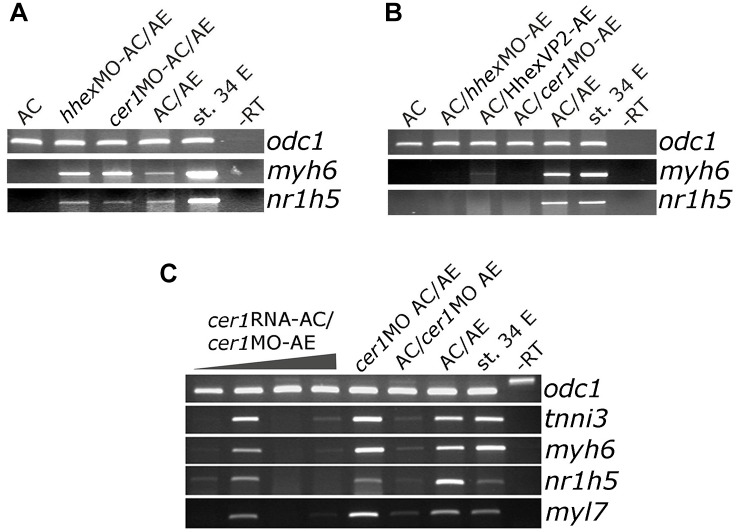
Cerberus and *hhex* are required in anterior endoderm for liver and cardiac specification. **(A)** Injection of *cer1* or *hhex* MOs (10 or 20 ng/embryo, respectively) in AC have no effect on heart and liver marker expression in AC/AE conjugates. This is in contrast to **(B)**, where injection of *cer1* or *hhex* MOs in AE leads to downregulation of both cardiac and liver marker gene mRNA levels in conjugates. Injection of mRNA (500 pg/embryo) coding for dominant negative Hhex-VP2 construct had the same effect as *hhex* MO. **(C)** Cardiac and liver specification deficiency in AC/*cer1* MO-AE conjugates can be rescued by Cerberus. c*er1* mRNA was injected in AC at increasing concentrations, from left to right; 100, 200, 500, and 1000 pg per embryo. All samples were cultured until stage control had reached stage 34 where PCR was carried out for indicated markers.

*Cerberus* (*cer1*) and *hhex* have both been shown to be required for normal development of the anterior end of the embryonic axis and for cardiac and AE specification (Brickman et al., 2000; Martinez Barbera et al., 2000; Foley and Mercola, 2005; Foley et al., 2007). We took advantage of the AC/AE model to specifically downregulate *hhex* or *cer1* in AC (Figure 3A) or AE (Figure 3B) using previously described MOs against *cer1* (Kuroda et al., 2004) and *hhex* (Smithers and Jones, 2002). We have confirmed effectiveness of *cer1*MO and *hhex*MO by showing that they affect heart development (Supplementary Figure S5). Our results demonstrate that both *hhex* and *cer1* are specifically required in AE for cardiac and liver specification, in agreement with previous work (Foley and Mercola, 2005; Foley et al., 2007). Interference with Hhex function in AE by using dominant-negative construct HhexVP2 (Brickman et al., 2000) produced the same result as *hhex*MO (Figure 3B). Additional experiments have shown that *cer1* deficiency in the AE can be rescued by expression of *cer1* mRNA in AC, in a strict dose-dependent manner (Figure 3C).

### Differential Effect of Wnt/β Catenin Signaling Activation on Cardiac and Liver Specification

**FIGURE 4:**
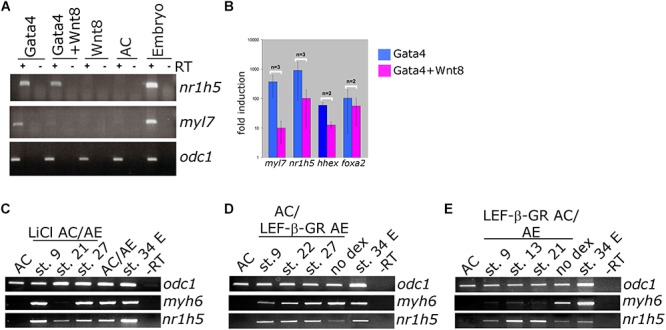
Differential effect of Wnt/beta catenin signaling activation on cardiac and liver cell fate. **(A)** Animal caps co-injected with Gata4 mRNA and Wnt8-expressing DNA show marked reduction of *myl7* RNA levels and negligible effect on *nr1h5* expression. **(B)** qPCR analysis confirms the findings of gel RT-PCR for *myl7* and *nr1h5* and in addition shows the reduction of *hhex* expression in Gata4 + Wnt8 samples. **(C)** AC/AE conjugates were treated with LiCl at indicated stages. Treatment at st. 21 blocked cardiac differentiation but had a lesser effect on liver specification. **(D)** Cell autonomous activation of Wnt/β-catenin signaling in LEF-β-GR mRNA-injected AE was achieved by adding dexamethasone (dex) to AC/AE conjugates at indicated stages. No effect on either heart or liver marker expression was observed. **(E)** In contrast, activation of LEF-β-GR in the AC part of the conjugates greatly reduced *myh6* expression without affecting *nr1h5*. Explants were collected for RT-PCR analysis when sibling embryo controls reached st. 34.

Wnt pathway activation has a well-documented attenuating effect on cardiac differentiation *in vivo*, in embryonic stem (ES) cell differentiation model and in Gata4-expressing AC from *Xenopus* embryos (Marvin et al., 2001; Schneider and Mercola, 2001; Tzahor and Lassar, 2001; Latinkic et al., 2003; Naito et al., 2006; Liu et al., 2007; Ueno et al., 2007). We have activated Wnt signaling in control animal cap explants or in those expressing Gata4 by zygotic co-expression of Wnt8. Wnt8 had no effect on its own on cardiac or liver markers but it attenuated cardiogenesis induced by Gata4 (Figure 4A,B; Latinkic et al., 2003). At the same time, the expression of the liver marker *nr1h5* was unaffected (Figure 4A,B).

We have previously shown that activation of Wnt signaling opposes cardiac differentiation but not specification in AC/AE conjugates (Samuel and Latinkic, 2009). Using the same experimental approach, we have activated Wnt/β-catenin signaling in AC/AE at different time points, either uniformly by LiCl (Figure 4C) or specifically in AC or AE by activation of an inducible chimaeric construct Lef-β-catenin-GR (LEF-β-GR, Figure 4D,E). Brief LiCl treatment causes a strong but transient activation of Wnt target genes *siamois* (*sia*) and *nodal3.1*, whose expression is undetectable 6 h after treatment, whereas activation of Lef-β-catenin-GR leads to a milder but sustained response (Samuel and Latinkic, 2009). Our results show that treatment with LiCl at an early time point, near the time of cardiac specification (st. 9) has no effect on either cardiac or liver specification, whereas treatment at the late neurula stage (st. 21) abolishes cardiac but not liver marker expression. Similarly, activation of Wnt signaling using Lef-β-catenin-GR in AC but not in AE affects cardiac but not liver markers.

**FIGURE 5:**
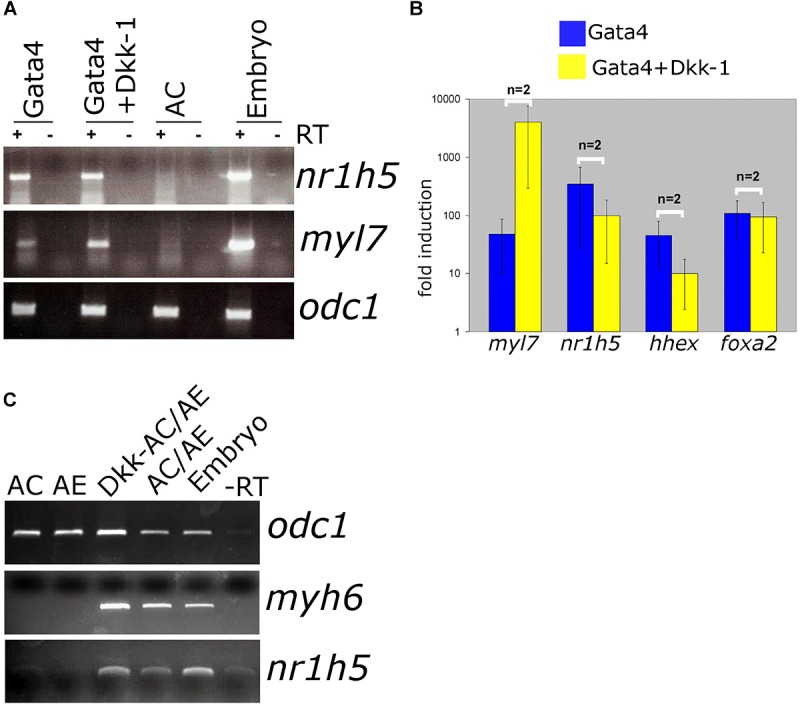
Wnt/β-catenin signaling is not required for liver specification in Gata4-AC and AC/AE. **(A)** Gata4 and *dkk-1* mRNA co-injection in animal pole explants causes a well-described increase in cardiac marker levels but has no effect on *nr1h5*. **(B)** qPCR analysis confirms these findings and extends them by showing no significant effect on *hhex* and *foxa2*. **(C)** *dkk-1* expression in AC/AE (injected in AC) likewise has no effect on *nr1h5*. RT-PCR analyses were performed on st. 34 explants and sibling embryo controls.

Antagonism of Wnt signaling is required for cardiac differentiation and has been shown to promote specification of AE-derived liver. In Gata4-expressing AC explants, antagonism of Wnt by secreted antagonist Dkk-1 has been shown to enhance cardiogenesis (Latinkic et al., 2003). At the same time, *nr1h5* expression is unaffected (Figure 5A,B). In AC/AE explants Dkk-1 does not affect cardio- and hepatogenesis (Figure 5C). Wnt was suggested to be required for liver bud outgrowth, suggesting that the AC/AE model does not capture those later stages of liver development.

### BMP Signaling Inhibition Attenuates Liver Cell Fate Specification

**FIGURE 6:**
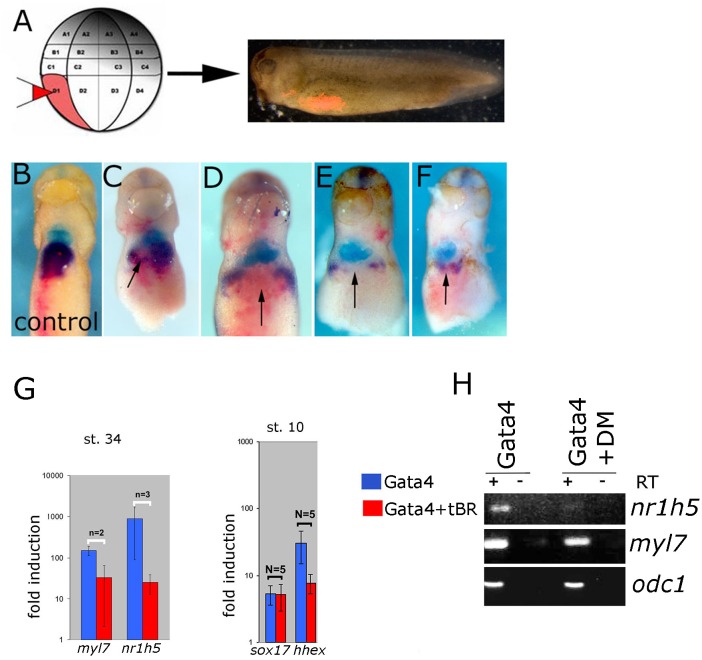
BMP signaling is required for liver development. **(A)** Design of the experiment. Lineage tracer was injected **(B)** alone or **(C–F)** with ∼30 pg/blastomere of truncated BMP Receptor (tBR) mRNA in dorso-vegetal blastomere D1 at the 32-cell stage. Heart and liver were revealed by double WMISH of *myl7* (turquoise) and *nr1h5* (purple). **(C–F)** four examples showing attenuation of liver fate specification *in vivo* following localized BMP inhibition by tBR (red-pink; pointed by arrows). *N* = 11, all showing effect on *nr1h5* expression. Ventral views are shown, anterior is up. BMP signaling inhibition attenuates liver cell fate specification in Gata4 injected AC. **(G)** qPCR analyses of st. 34 explants show downregulation of *nr1h5* as a consequence of BMP inhibition via tBR. At st. 10, tBR has no effect on the ability of Gata4 to induce *sox17* but reduces *hhex* induction. **(H)** Treatment of Gata4-expressing AC explants with 30 μM dorsomorphin (DM) leads to downregulation of *nr1h5*.

BMP signaling has been shown to be required for liver specification in a wide range of models (Gordillo et al., 2015). In agreement with this we show that cell autonomous inhibition of BMP signaling using truncated BMP receptor (BMPRI) targeted to foregut interferes with liver development in tadpoles (Figure 6A–F). We next wished to test the dependence of liver specification on BMP signaling in Gata4-induced hepatogenesis. Cardiogenesis induced by Gata4 in AC explants does not require BMP signaling (Latinkic et al., 2003; Figure 6G), however, inhibition of BMP signaling using truncated BMP receptor or a small molecule inhibitor Dorsomorphin lead to a decrease in expression of *nr1h5* at st. 34 and *hhex* at st. 10 (Figure 6G,H). These results suggest that BMP signaling is required for hepatic, but not cardiac, induction by Gata4 in pluripotent AC explants.

### FGF Signaling Is Not Required for Gata4-Mediated Liver Specification

**FIGURE 7:**
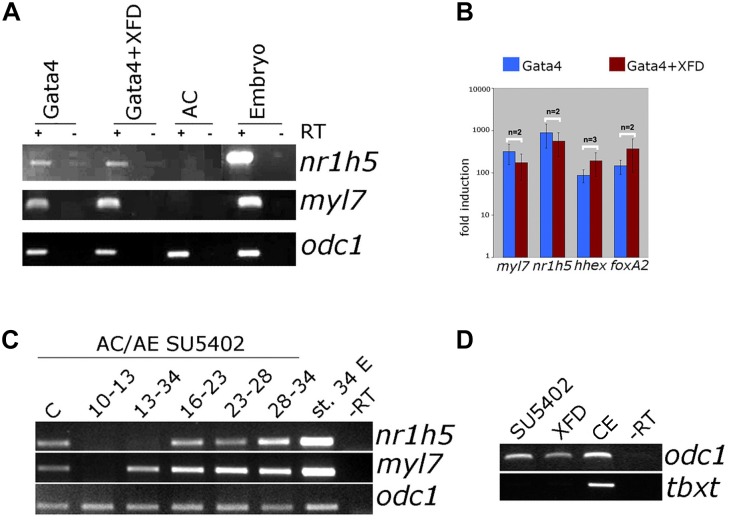
FGF signaling is not required for liver specification by Gata4 but is essential in AC/AE explants. **(A)** Gata4 and XFD (dominant-negative FGFR1) mRNA co-expression in animal pole explants has no effect on *nr1h5* and *myl7* expression. **(B)** qPCR analysis confirms these findings and extends them by showing no significant effect on *hhex* and *foxa2*. **(C)** Treatment of AC/AE with FGFR inhibitor SU5402 (50 μM) has stage-specific effect. Immediately upon AC/AE conjugate formation, SU5402 inhibits cardiac and liver gene expression. Treatment from st. 13 until the end of incubation at st. 34 has no effect on cardiogenesis but inhibits liver specification. Shorter time windows of treatment are largely without effect. Staging is according to the stage of AE (st. 10 at the time of conjugation). C-Control explants treated with 50 μM DMSO. **(D)** SU5402 and XFD are effective inhibitors of early mesodermal marker and FGF target gene *tbxt*. CE-control (untreated) sibling embryos. RT-PCR analyses were performed on st. 34 explants and sibling embryo controls.

FGF signaling has a well-documented role in liver specification (Zaret, 2016). We have next examined the involvement of FGF signaling in liver specification in Gata4-expressing AC explants. Downregulation of the FGF pathway using dominant-negative FGFR1 (XFD; Figure 7A,B) or small drug SU5402 (Supplementary Figure S3) has no effect on both liver and cardiac specification, suggesting that the FGF pathway is not required in Gata4-induced liver cell fate specification in AC explants. Effectiveness of SU5402 and XFD was shown by their ability to inhibit expression of early mesodermal marker and FGF target *tbxt* (formerly *xbra*; Figure 7D) and by induction of characteristic gastrulation defect phenotype (Supplementary Figure S4). In AC/AE explants, the FGF pathway is required for cardiogenesis immediately following formation of conjugates (Samuel and Latinkic, 2009; Figure 7C). Not surprisingly, under these conditions liver specification is also affected. In contrast, inhibition of the FGF pathway from st. 13 until the end of culturing period at st. 34 had no effect on cardiac differentiation but attenuated *nr1h5* expression (Figure 7C). Shorter treatment time windows (st. 16–23, 23–28, 28–34) had no major effect on cardiac and liver cell fate specification (Figure 7C).

## Discussion

In this report we have used two experimental models based on *Xenopus* embryos that permit induction of liver and cardiac fates, to investigate their specific signaling requirements.

The simpler of the models is based on Gata4-mediated induction of liver specification in pluripotent animal cap explants. In this model, when Gata4 mRNA is expressed throughout the explant, both cardiac and liver cell fates are induced, suggesting that fate acquisition is stochastic under conditions when Gata4 is most likely acting cell-autonomously. In comparison, a bona fide endoderm determinant Sox17 induces liver gene expression in animal pole explants only non-cell autonomously. It would be of interest to further explore cell autonomous mode of Gata4 action in liver specification in animal cap explants.

The second model used in the current study is based on heterochronic conjugates of gastrula-stage AE and blastula-stage animal cap explants. In these AC/AE conjugates AE induces cardiac specification in the overlaying animal cap ectoderm (Samuel and Latinkic, 2009), and in this study we have shown that AC/AE conjugates also express a range of endoderm markers. The liver tissue in conjugates is induced in the endoderm, adjacent to the cardiac domain which has been induced in the animal cap explant (Figure 1). This configuration closely resembles the spatial relationship of the heart and the liver in the early embryo and suggests that AC/AE conjugates recapitulate many aspects of cellular and molecular interactions that govern cardiac and liver specification in the embryo. In addition to the liver markers, AC/AE conjugates expressed a marker of lung and thyroid, *nkx2-1*, as well as subset of pancreatic markers-*pdia2* and *pdx1*, but not *ins*, suggesting that partial reprogramming toward endocrine pancreas has been achieved by st. 43.

We have used the AC/AE model to examine the roles of *hhex* and *cer1* in AC or AE. As expected (Foley and Mercola, 2005), *hhex* was found to be required in the AE for cardiac as well as for liver specification (Figure 3). Similarly, *cer1* is required in the AE (Figure 3), in agreement with the findings by Foley et al. (2007). The deficiency of *cer1* in AE can be rescued by injection of cer1 mRNA in the AC, but only in a narrow concentration range, suggesting that the pathways regulated by the Cerberus protein, BMP, Wnt, and Nodal (Piccolo et al., 1999), operate at a finely tuned level. In future it would be of interest to use AC/AE conjugates to dissect the requirement of each of these pathways, for example by asking whether and when Cerberus function could be replaced by small molecule inhibitors of Wnt, BMP and Nodal pathways, as well as to examine epistatic relationship between *hhex* and *cer1*.

Wnt pathway activation interferes with cardiogenesis both in Gata4-expressing animal cap explants and in AC/AE conjugates (Latinkic et al., 2003; Samuel and Latinkic, 2009), but it does not significantly affect liver specification in both models (Figure 4). In Gata4-expressing AC explants liver specification is apparently independent of the presence of differentiated cardiomyocytes. One possibility is that inductive signaling between cardiac mesoderm and endoderm in this model occurs prior to cardiac differentiation and the second one is that liver cell fate is induced cell autonomously by Gata4.

We have previously shown that expression of cardiac precursor markers *nkx2-5* and *tbx5* is not affected by Wnt activation in AC/AE conjugates, suggesting that cardiac precursors transiently produce a liver-inducing signal. Under the conditions of Wnt pathway activation cardiac precursor are prevented to undergo differentiation into cardiomyocytes, showing that the production of the liver-inducing signal does not require cardiomyocytes and that the liver-inducing signals are likely transiently produced by cardiac progenitors.

Several studies have shown that Wnt antagonism is required for liver specification (Zorn and Wells, 2009; Zaret, 2016). Most relevant for the current study is the work by McLin et al. (2007) who have shown that Wnt antagonizes foregut development in *Xenopus* embryos. The apparent discrepancy between the two studies is likely due to the differences between the models that were used, *in vivo* by McLin et al. and explants in the current study. In animal cap explants Gata4 might be acting in parallel to or downstream of *hhex* in specifying liver, as *hhex* has been shown to be downstream of Wnt antagonism (Foley and Mercola, 2005).

Our results have suggested that BMP signaling is required for liver specification downstream of Gata4. It will be of interest to examine in more detail how and when BMP antagonism interferes with Gata4-induced hepatogenesis. In addition we have found that BMP signaling is required for liver specification *in vivo*, as previously reported (Zorn and Wells, 2009; Zaret, 2016).

Unlike BMP inhibition, interference with FGF signaling was found to have no effect on Gata4-driven liver cell fate specification in animal cap explants (Figure 7 and Supplementary Figure S3). In contrast, liver specification in AC/AE explants shows requirement for prolonged FGF signaling (st. 13–34), but shorter treatment windows during neurula and tailbud stages show no effect (Figure 7). This finding suggests that FGF signaling is required for liver specification over a prolonged period rather than within a discrete, well-defined time window. Early FGF inhibition immediately after conjugation of AC/AE explants inhibits cardiogenesis and this likely leads to an inhibition of liver specification as well.

Our results with AC/AE explant conjugates are in good overall agreement with those of Shifley et al. (2012) who reported that prolonged FGF signaling is required for liver specification in *Xenopus* embryos. Taken together with the results with manipulation of Wnt signaling, our findings suggest that cardiac precursors produce a liver specifying signal, most likely an FGF, which is required over a prolonged period to specify liver fate.

The principles of liver specification and differentiation that were established in various vertebrate embryos have been and are the key to the development and refinement of protocols for liver cell differentiation from pluripotent stem cells. This is an area of intense research due to potential medical applications and our findings may inform future attempts at refinement of differentiation protocols.

## Author Contributions

KH performed most of the animal cap experiments. LS performed most of the AC/AE conjugate experiments. SB and PK contributed to analysis of several experiments. BL contributed to experimental manipulation of embryos, planned the study, and wrote the manuscript.

## Conflict of Interest Statement

The authors declare that the research was conducted in the absence of any commercial or financial relationships that could be construed as a potential conflict of interest.

## Footnotes

1 http://www.gene-tools.com/
