# Supplementary figures and images for: Liver Specification in the Absence of Cardiac Differentiation Revealed by Differential Sensitivity to Wnt/β Catenin Pathway Activation
Source: Front Physiol. 2019 Mar 5;10:155. doi: 10.3389/fphys.2019.00155 (PMC6411699; doi:10.3389/fphys.2019.00155)

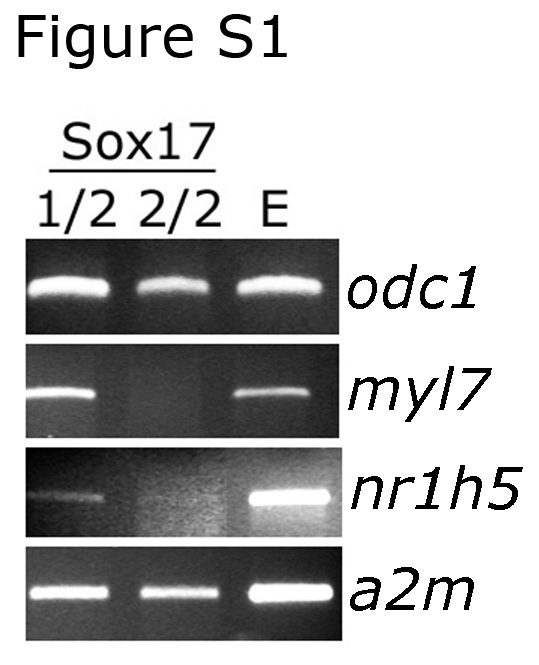

Supplement: Supplementary file 1 [file Image_1.JPEG]

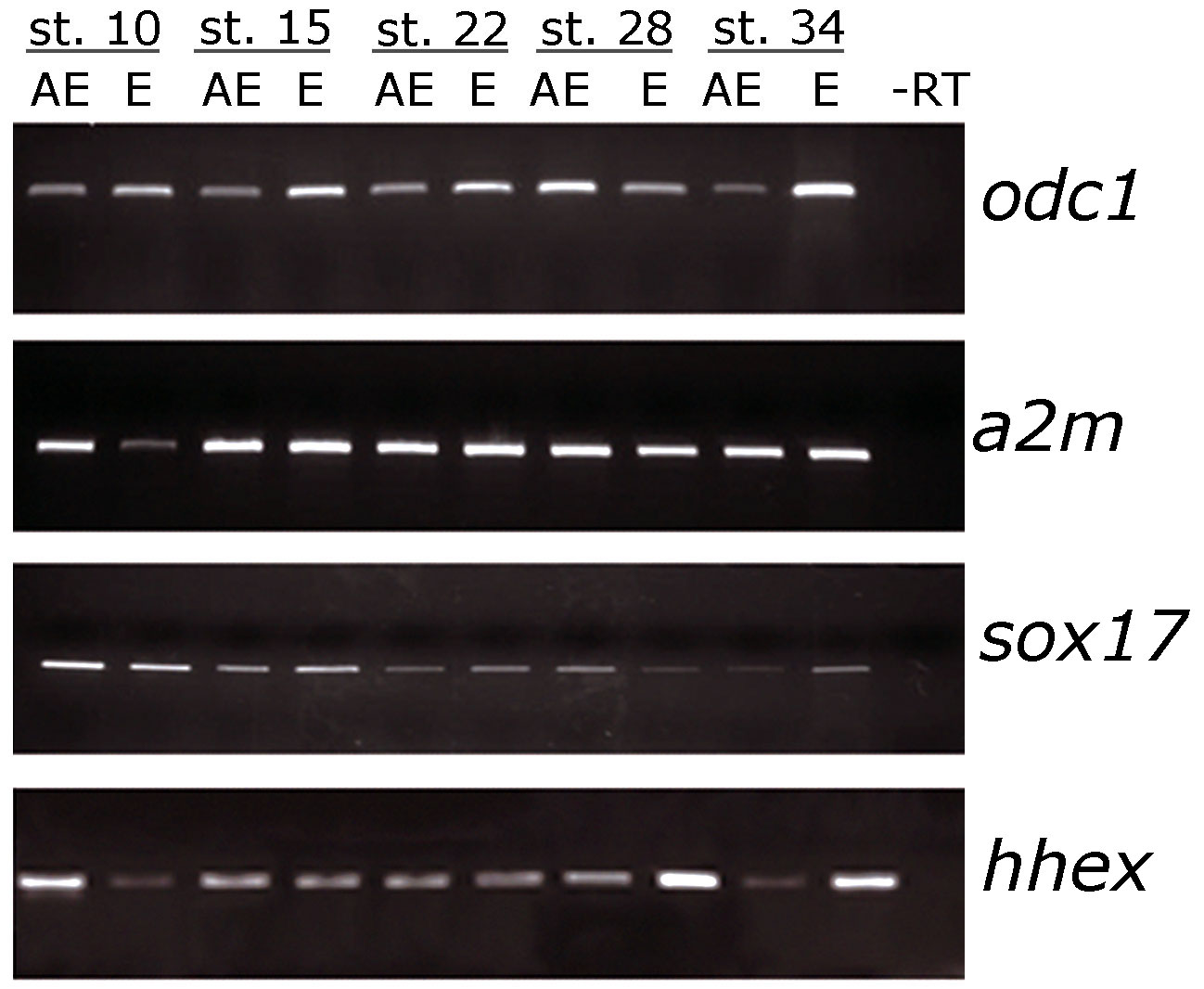

Supplement: Supplementary file 2 [file Image_2.JPEG]

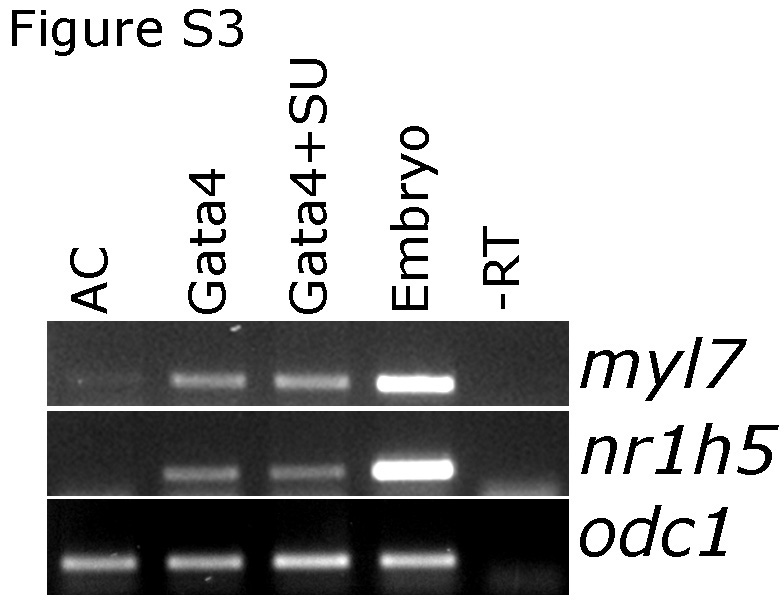

Supplement: Supplementary file 3 [file Image_3.JPEG]

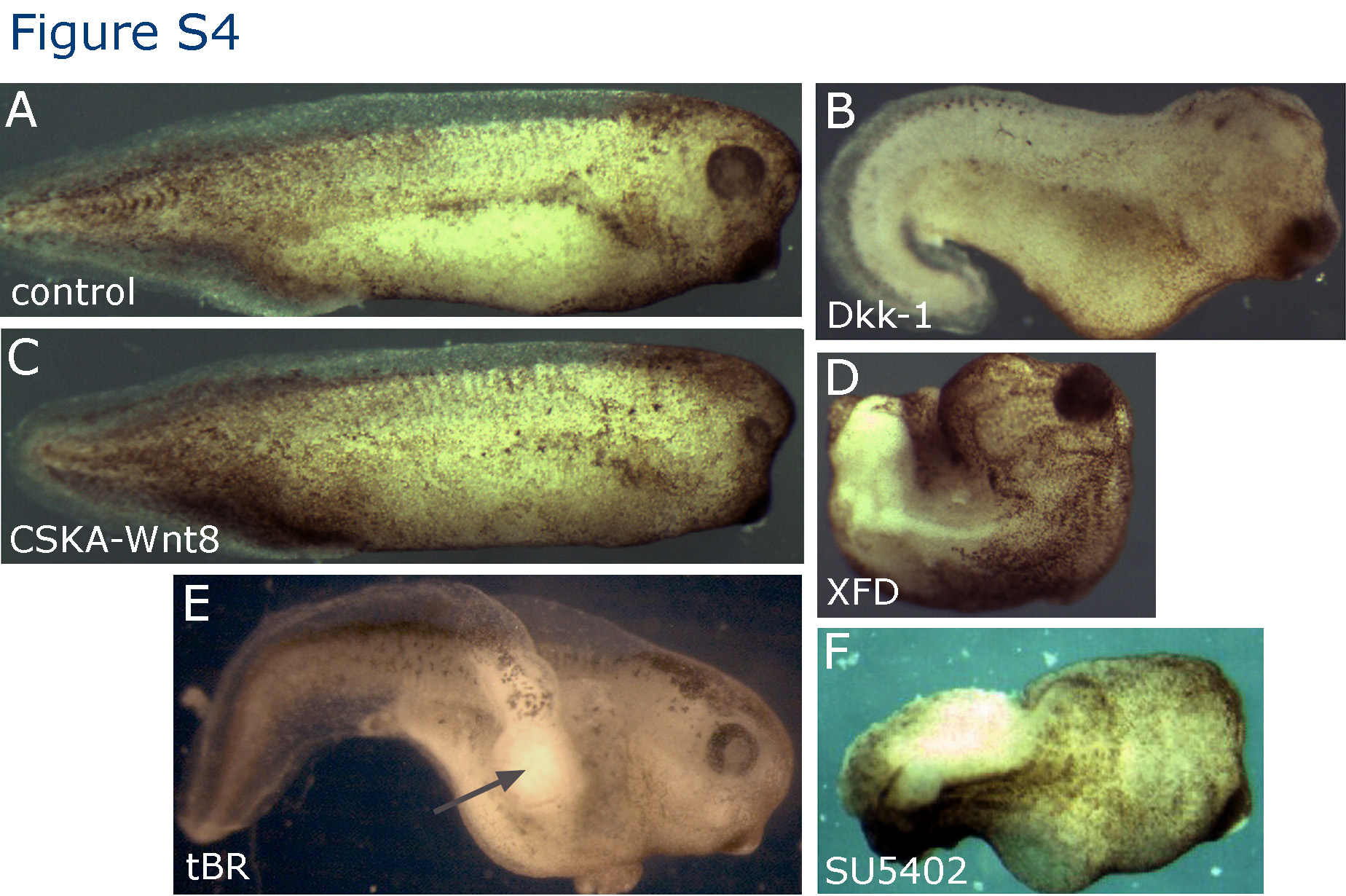

Supplement: Supplementary file 4 [file Image_4.JPEG]

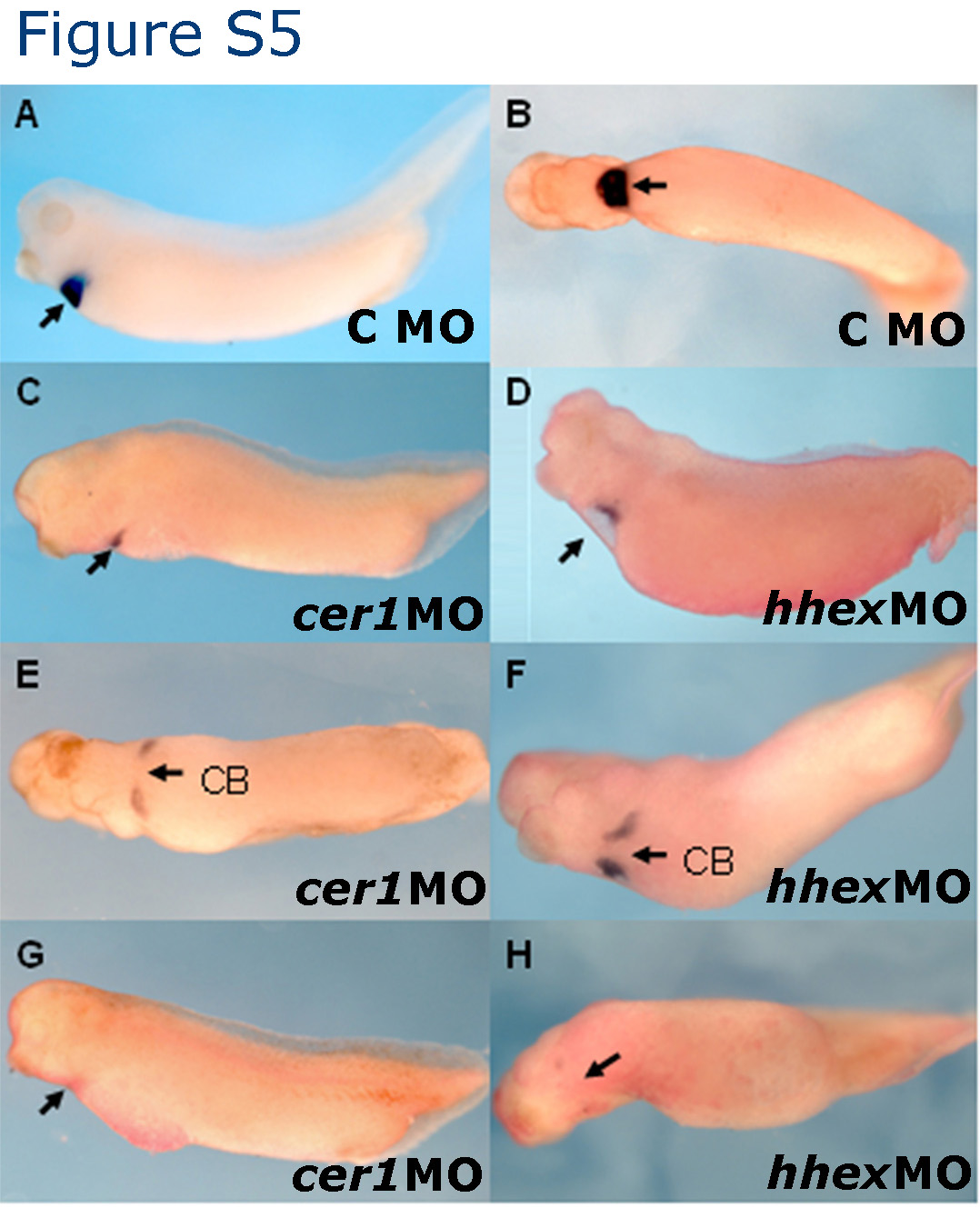

Supplement: Supplementary file 5 [file Image_5.JPEG]
