# Supplementary material for: Liver Specification in the Absence of Cardiac Differentiation Revealed by Differential Sensitivity to Wnt/β Catenin Pathway Activation
Source: Front Physiol. 2019 Mar 5;10:155. doi: 10.3389/fphys.2019.00155 (PMC6411699; doi:10.3389/fphys.2019.00155)
Supplement: Supplementary file 6 [file Table_1.DOCX]

**Figure S1. Non-cell autonomous induction of liver cell fate by Sox17.** Animal cap explants injected with *sox17* mRNA in one (1/2) or both blastomeres (2/2) at the 2-cell stage were cultured until sibling control embryos (E) reached st. 34 and were analysed for expression of indicated markers by RT-PCR. Whilst both uniformly- and hemi-injected embryos express endoderm marker *a2m* (endodermin), only hemi-injected explants also express *nr1h5*.

**Fig. S2. Anterior Endoderm explants retain endodermal character and maintain expression of *hhex***. AE explants excised at st. 10 were cultured until indicated stages and together with stage control embryos (E) were processed for RT-PCR. AE explants maintain endodermal character (*a2m*, *sox17* expression) at all stages examined. Expression of *hhex* is enriched in st. 10 AE, attesting to anterior character of explants.

**Fig. S3. Inhibition of FGF pathway by SU5402 has no effect on Gata4-induced expression of *myl7* and *nr1h5* in AC explants**. Treatment with 20 μM SU 5402 was immediately following excision of AC explants at st. 9 until the end of incubation at st. 34. Control AC explants were treated with 20 μM DMSO.

**Fig. S4.** **Phenotypic controls for effectiveness of reagents for manipulation of signalling pathways used in this study.** Embryos were treated as indicated in the panels and were analysed at st. 34-37. Representative samples from at least 20 for each treatment are shown. (A) control untreated embryo. (B) Dkk-1 mRNA injected embryo, showing prominent anteriorisation. (C) CSKA-Wnt8 DNA injected embryo, showing characteristic posteriorisation (reduction of eyes and head). (D) XFD (dominant negative FGFR1) mRNA injected embryo, showing gastrulation defect affecting the trunk and tail, with head being less affected. (E) tBR (truncated BMPR) injected embryo. Injection in 1 ventral blastomere at 4-cell stage leads to a partial secondary axis (arrow), characteristic of dorsalising activity of tBR. (F) SU5402 treated embryo, showing a phenotype similar to the XFD injected embryo. Anterior is to the right in all panels.

**Fig. S5. Knockdown of Cer1 and Hhex leads to defective heart development.** Representative examples of phenotypic classes of embryos injected with indicated morpholino oligonucleotides and processed for expression of *myl7* at st. 36 are shown. (A, ,B) Control MO (C MO) injected embryos show normal *myl7* expression (n=25). Cardiac phenotype for *cer1*MO samples (*n*=59): 85% reduced *myl7* expression (C), 6% split (E) and 9% absent (G); for *hhex*MO samples (*n*=48): 64% reduced (D), 14% split (F; Cardia Bifida, CB) and 22% absent (H). Anterior is to the left. B, E, F, H: ventral view. A,C, D, G: lateral view.
